# The Roles and Pathogenesis Mechanisms of a Number of Micronutrients in the Prevention and/or Treatment of Chronic Hepatitis, COVID-19 and Type-2 Diabetes Mellitus

**DOI:** 10.3390/nu14132632

**Published:** 2022-06-24

**Authors:** Khalid M. Sumaily

**Affiliations:** Clinical Biochemistry Unit, Department of Pathology, College of Medicine, King Saud University, Riyadh P.O. Box 145111, Saudi Arabia; ksumaily@ksu.edu.sa

**Keywords:** trace elements, micronutrients, hepatitis, COVID-19, diabetes, zinc, copper, magnesium, selenium, iron

## Abstract

A trace element is a chemical element with a concentration (or other measures of an amount) that is very low. The essential TEs, such as copper (Cu), selenium (Se), zinc (Zn), iron (Fe) and the electrolyte magnesium (Mg) are among the most commonly studied micronutrients. Each element has been shown to play a distinctive role in human health, and TEs, such as iron (Fe), zinc (Zn) and copper (Cu), are among the essential elements required for the organisms’ well-being as they play crucial roles in several metabolic pathways where they act as enzyme co-factors, anti-inflammatory and antioxidant agents. Epidemics of infectious diseases are becoming more frequent and spread at a faster pace around the world, which has resulted in major impacts on the economy and health systems. Different trace elements have been reported to have substantial roles in the pathogenesis of viral infections. Micronutrients have been proposed in various studies as determinants of liver disorders, COVID-19 and T2DM risks. This review article sheds light on the roles and mechanisms of micronutrients in the pathogenesis and prevention of chronic hepatitis B, C and E, as well as Coronavirus-19 infection and type-2 diabetes mellitus. An update on the status of the aforementioned micronutrients in pre-clinical and clinical settings is also briefly summarized.

## 1. Introduction

Trace elements (TEs), also known as trace metals, are chemical elements with a concentration (or other measures of an amount) that is very low [1]. TEs are constituents of every living organism and despite being present in relatively small amounts, they play vital roles in the growth, development as well as the general well-being of the body’s organisms [2]. The immune system requires a high supply of certain TEs to accomplish the essential functions needed in defence and surveillance [2,3,4,5,6,7]. Some vitamins and trace elements (TEs) are reported to have key roles to cope with viral infections, such as chronic hepatitis [2,3,4,5,6,7], and COVID-19 [8,9,10], in addition to metabolic disorders, such as type-2 diabetes mellitus (T2DM) [9,11,12].

The essential TEs, such as copper (Cu), selenium (Se), zinc (Zn) and iron (Fe), are among the most commonly studied micronutrients [13]. While every element has its own distinctive role in human health, TEs, such as iron (Fe), zinc (Zn) and copper (Cu), are considered essential for the organisms’ well-being due to their involvement in several metabolic pathways where they play roles as enzyme co-factors, anti-inflammatory and antioxidant agents [14]. The homeostasis of essential trace elements is maintained by the liver [15]. Therefore, an impaired liver function usually results in certain disturbances in the TEs metabolism, which leads to initiating oxidative stress and consequently results in inflammatory and/or fibrotic alterations in the liver [4].

Hepatitis is a viral infection that is characterized by an inflammation in the liver. Although this inflammation could develop concurrently or result from alcoholic and non-alcoholic steatosis, it can also be caused solely by a viral infection [16]. Recently, the impaired metabolism of a number of trace elements has been shown to have a role in the hepatitis virus infection process. Some trace elements, such as copper (Cu), zinc (Zn), selenium (Se) and iron (Fe), were shown to play a direct role in the infection and the immune rejection against hepatitis C virus (HCV) [17].

Studies have been recently conducted and indicated the benefits of using micronutrients as a natural approach to manage the infectious diseases that affect the respiratory system [18,19,20,21]. Given the current COVID-19 pandemic, which has a high mortality rate, particularly in critically ill patients [12], many studies have been conducted to distinguish the different clinical symptoms; however, the data concerning its pathology as well as the cellular responses to this virus are still limited [22]. 

Therefore, studies with different interventions have been recently conducted to manage COVID-19, which includes the studying and assessment of micronutrients and their roles in this viral infection [23]. Dietary management was recently proposed as a strategy that minimizes the potential risks of COVID-19 infection [24]. The adjuvant supply of certain important micronutrients that function as positive modulators in the immune system was shown to provide a further support to this strategy, in which some vitamins, such as vitamin A, B6, B12, C, D and E, as well as some essential trace elements, including zinc (Zn), iron (Fe), selenium (Se) and copper (Cu), as well as electrolytes, such as magnesium (Mg), are considered promising [25].

On the other hand, trace element imbalances may have a negative impact on biological processes that are related to fatal diseases, such as type-2 diabetes mellitus (T2DM) [26]. Studies have been performed to evaluate the relationship between the serum levels of various trace elements and metabolic disorders, such as T2DM [2,23]. Many studies have observed a direct association between a number of micronutrients and type-2 diabetes mellitus. 

Some micronutrients, such as chromium (Cr), magnesium (Mg), vanadium (V), zinc (Zn), manganese (Mn), molybdenum (Mo) and selenium (Se), were shown to potentiate the insulin action by reducing the levels of blood glucose [26,27]. A number of mechanisms were proposed in regard of the potentiation of insulin by trace elements, and these include activating insulin receptor sites, acting as cofactors for some enzyme involved in the metabolism of glucose, increasing the sensitivity of insulin and exerting their antioxidant effects to prevent tissue peroxidation [28]. 

The Diabetes Prevention Program (DPP) has shown that a change in lifestyle, (i.e., weight loss, weight maintenance and physical activity) can decrease the incidence of T2DM by 58% [29]. However, implementing these interventions in the real life has been shown to be problematic. Thus, it is urgently needed to discover other preventive interventions that can be provided as real-world solutions [30]. Advances in the techniques used in molecular biology have opened the doors for researchers to provide further clarification to the novel mechanisms of TEs that cause these metabolic abnormalities [27]. 

Despite the recent reviews that have assessed the underlying mechanisms of TEs in the prevention and/or development of metabolic abnormalities, clinical studies and laboratory analyses are still few, and the database available is limited [13]. Table 1 provides a summary of the pathogenic pathways and roles of a number of micronutrients in the prevention and/or treatment of chronic hepatitis, COVID-19 and T2DM [28,29,30,31,32,33,34,35,36,37,38,39,40,41,42,43,44,45,46,47,48,49,50,51,52,53,54,55,56,57,58,59]. This review article assesses the roles and the importance of a number of micronutrients in chronic hepatitis, COVID-19 and T2DM, while giving a brief summary on the therapeutic and preventive mechanisms as well as the current status of zinc (Zn), selenium (Se), copper (Cu), iron (Fe) and magnesium (Mg) in preclinical and clinical settings.

## 2. Roles and Mechanisms of Trace Elements in Viral Infections

The epidemics of infectious diseases are becoming more frequent and spreading at a faster pace around the world, which results in major impacts on the economy and health systems [62]. Different trace elements were reported to have different roles in the pathogenesis of viral infections, including the viruses’ survival, the attachment to their host as well as the characterization of the viral infections that occur as a result of deregulating metal homeostasis in the course of infection [9]. For instance, many studies have revealed that zinc (Zn) [8], copper (Cu) [10], and iron (Fe) [63], are some of the metals that commonly bind to the proteins that were shown to have an association in the occurrence of viral infections [21]. Therefore, this section highlights the biochemical mechanisms and roles by which these trace elements act in the immune system against chronic hepatitis and COVID-19 viral infections in particular.

### 2.1. Roles and Mechanism of Trace Elements in Chronic Hepatitis

It is believed that the replication of the virus is considered as the driving force in viral infections that cause liver damage [64]. International guidelines have shown that the primary therapeutic goal in the treatment of chronic Hepatitis B virus (HBV) is to permanently suppress the replication of the virus [65]. Some publications have reported that serum concentration of trace elements, such as zinc (Zn), selenium (Se), copper (Cu) and iron (Fe), showed a high sensitivity for assessing hepatic disorders [66]. In addition, studies have shown that these aforementioned trace elements play key roles in liver diseases and more specifically degenerative liver disorders [67].

Although the plasma concentrations of these trace elements were shown to change during the majority of infections, it is still unclear whether the infected tissues (i.e., the liver) experience the same alteration [65]. It is believed that the activity of HBV is altered by the change of the serum levels of these major trace elements [19]. However, the variation of plasma levels of trace elements in viral infections and their effect on creating a tissue injury is still unknown [3].

The mechanisms by which viral infections cause liver damage are presented through extensive inflammation and oxidative stress, which result from producing excessive reactive oxygen species (ROS) [68]. This section highlights a number of studies that addressed the relationship between the development of chronic hepatitis and the effects that some trace elements, such as zinc (Zn), selenium (Se), iron (Fe) and copper (Cu), may have in the different types of hepatitis viral infection.

#### 2.1.1. Zinc (Zn)

Zinc (Zn) is an essential trace element that was reported to increase the susceptibility of developing many diseases due to its involvement in various metabolic processes that have a vital role in the immune system [69]. Liver is the organ that has the main responsibility of metabolizing zinc (Zn) [7]. Studies have shown that the levels of serum zinc (Zn) are often lowered in patients who have chronic liver diseases [70]. Other studies have shown that zinc (Zn) deficiency altered the functions of hepatocytes as well as the immune responses in some inflammatory liver diseases [71], which as a consequence, initiated some metabolic abnormalities that include insulin resistance [72], iron (Fe) overload [11], hepatic steatosis [73], and hepatic encephalopathy in patients suffering from a chronic liver disease [74].

On the other hand, data collected on the zinc (Zn) supplementation effect were conflicting [69], and even though the use of zinc (Zn) supplementation has been shown to help in alleviating some of the symptoms detected in chronic liver diseases, in addition to the positive effects it had on metabolic abnormalities as reported in some experimental models, there is still no strong evidence confirming the beneficial effects of using zinc (Zn) supplementation for patients with liver cirrhosis [75].

Previous studies have suggested that the antiviral state that activates the innate immune response involved the down-regulation of HBV mRNA [43,44,76]. In a study, it was demonstrated that the two human zinc finger antiviral protein (hZAP) isoforms (i.e., h-ZAP S and h-ZAP L) inhibited the replication of HBV in human hepatocyte-derived cells via the viral pgRNA posttranscriptional down-regulation [77]. In a mechanistic manner, the zinc finger motif-containing N-terminus of hZAP has the responsibility of reducing HBV RNA, as well as altering the four zinc finger motifs’ integrity in order to facilitate the binding of ZAP to HBV RNA and therefore fulfil the expected antiviral function [78].

The results of a similar study demonstrated that an upregulation of ZAP in cultured primary human hepatocytes as well as hepatocyte-derived cells was detected as a result of either IFN-α treatment or the activation of IPS-1. Knock-down of the expression of ZAP was shown to increase the HBV RNA levels and cause a partial attenuation of the antiviral effect stimulated by IPS-1 in the cell cultures used [31]. Consequently, ZAP was demonstrated as an intrinsic host antiviral factor that is active against HBV and plays a major role in the HBV replication innate control [79].

Another study has focused on analysing the roles and mechanisms of zinc (Zn) in the pathogenesis of hepatitis C virus (HCV) and have reported that the oxidative stress causes a disruption in zinc (Zn) homeostasis, in particular, in the signalling molecule and secondary messenger through the redox process, resulting in HCV-mediated mitochondrial dysfunction [80]. It is worth mentioning that, upon viral eradication using interferon (IFN)-based regimens or direct-acting anti-viral (DAA) therapy, zinc (Zn) serum levels were shown to rise significantly, which reflects the hepatic inflammation’s resolution as well as an improvement in the gut absorption [33].

**Figure 1:**
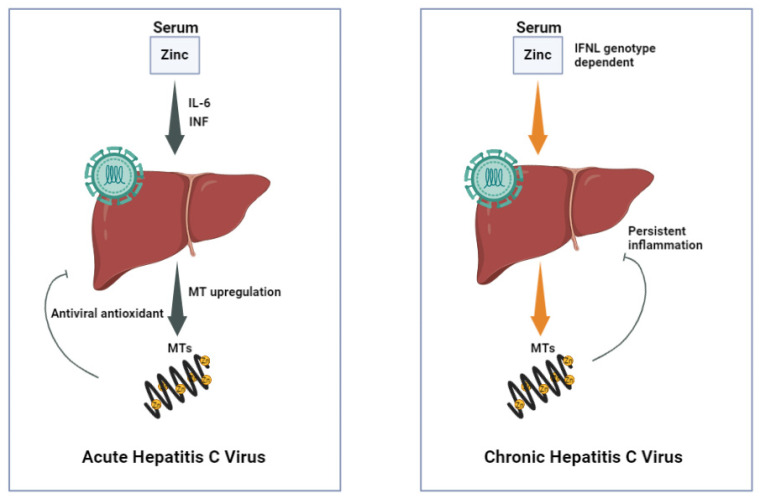
Roles of serum zinc and methallothionein in acute versus chronic hepatitis C virus (HCV).

Similarly, it was demonstrated in another study that a larger decline in the serum levels of zinc (Zn) following an IFN-α treatment resulted in an increase in the baseline zinc (Zn) levels, which in such conditions, zinc (Zn) was shown to mainly localize in the liver, causing a stimulation of the metallothionein (MT) expression and the anti-viral activity. The roles of serum zinc (Zn) and methallothionein in acute versus chronic hepatitis C virus (HCV) [4], are shown in Figure 1.

IFN-L3 is a proinflammatory cytokine, which has shown a potent antiviral activity against both acute and chronic infections [81]. In a recent study, zinc (Zn) was found to cause an inhibition of the IFN-L3 binding to its receptor (IFNLR1). The results demonstrated that zinc (Zn) enhanced the replication of both HCV as well as H1N1 (human influenza) in mammalian cells by causing an inhibition of the IFN-L3 function [82]. Reduced plasma zinc (Zn) levels and a reduction in the hepatic metallothionein (MT) expression were both detected in chronic HCV infection [34].

Therefore, studies have suggested that the mediated modulation of zinc (Zn) in the IFN-l signalling pathway, has been shown to play a significant role in the determination of disease outcome in patients with HCV [82,83]. A study that was conducted recently has analysed the antiviral action of zinc (Zn) in patients with hepatitis E virus (HEV) infection. In this study, zinc (Zn) treatment was shown to significantly inhibit the replication of the virus in the human hepatoma cell culture of genotype-1 and genotype-3 HEV. The results demonstrated that adding zinc (Zn) has inhibited the RNA-dependent RNA polymerase (RdRp) viral activity in vitro [7]. However, these observed data of the antiviral effect of zinc (Zn) could be related to direct and indirect actions of this trace element on multiple virus or host targets or processes [83].

#### 2.1.2. Selenium (Se)

Selenium (Se) has been studied for its involvement in the liver pathology and studies have reported that a deficiency in the serum levels of selenium (Se) was shown to induce a systematic redox imbalance as well as an inflammation in the blood [84]. Several selenoproteins including thioredoxin reductases (TXNRD), selenoproteins P (SELENOP), SELENOS and SELENOK and glutathione peroxidases 1 (GPX1) were studied and the results demonstrated that selenocysteines have a unique chemical reactivity and the ability to repair and mitigate liver damage caused by reactive oxygen species (ROS) [85].

Recently, an epidemiological study was conducted to assess the relationship between the serum levels of selenium (Se) and the risk of developing chronic liver diseases. This study reported that selenium (Se) serum levels are decreased when Se is at the optimal level in patients with hepatitis, cirrhosis and liver cancer as compared to healthy individuals [86]. However, these results were not shown to be consistent and the relationship in chronic liver diseases with different severities was controversial [87].

Furthermore, studies reported that a maintenance of an adequate amount of selenium (Se) in the body or the use of selenium (Se) supplementation in the case of selenium (Se) deficiency could have beneficial effects on patients who have chronic liver diseases, when compared with the controls in the same region [68,88]. In the case of CHC, the effect of selenium (Se) deficiency remains unclear. CHC infection has been reported in some studies to cause a reduction in the serum levels of selenium (Se), which have also been shown to decrease even more after the development of HCV-related cirrhosis [35]. These data were further supported by other studies, which have reported a decline in serum levels of selenium (Se) in proportion to the hepatic fibrosis degree [89].

In addition, the activity of glutathion peroxidase (GPx) was shown to reduce along with the serum levels of selenium (Se) in patients with CHC, which uncovered a possible mechanism for stimulating oxidative stress by CHC due to the deficient selenium (Se) levels [36]. However, the reduction of serum selenium levels in CHC patients was shown to be insignificant, which suggested that the alcohol consumption is the major variable affecting selenium (Se) levels [4]. HCV was reported in an in vitro study to inhibit gastrointestinal-GPx expression, which resulted in an increase in the replication of the virus [90].

**Figure 2:**
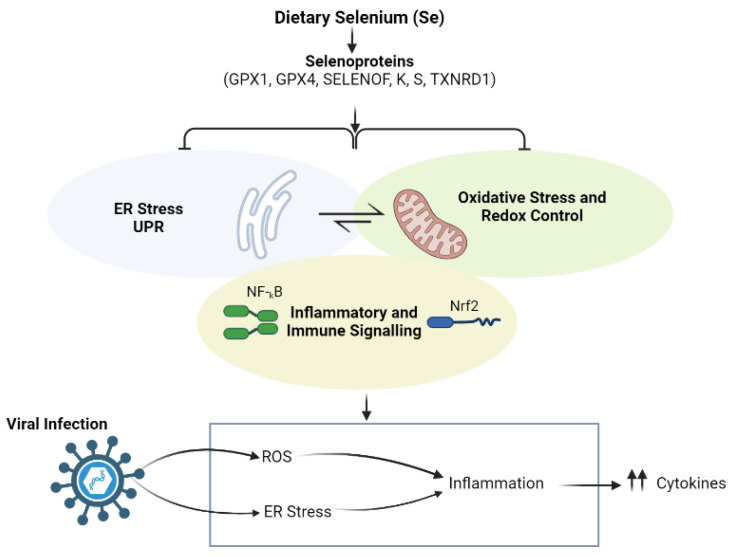
The role of dietary selenium (Se) in viral infections.

In addition, low serum selenium (Se) levels that were reported in CHC patients were shown to have a positive association with the lowered GPx activity; however, this observation has not been detected in HCV genotype or HCV-RNA load [91]. While these data do not give a direct indication that HCV results in a decreased selenium (Se) level, they still are raising the possibility to replace selenium (Se) as a therapeutic supplement in order to boost anti-oxidant as well as antiviral activities [4]. The role of dietary selenium (Se) in viral infections [92], is represented in Figure 2.

#### 2.1.3. Iron (Fe)

An enhancement in the progression of chronic HBV infection along with the patient’s poor prognosis were related to the high iron (Fe) serum levels [37]. In a study that was conducted in 2018, Gao et al. found that the levels of serum iron (Fe) and serum ferritin were increased in patients with chronic hepatitis B (CHB). It was observed in the same study that the levels of serum transferrin and the total binding capacity decreased while the transferrin saturation increased [38]. Other studies suggested that some inflammatory factors, such as liver injury, micro ribonucleic acid −122, viral activity, ROS, IL-6 as well as other factors could result in an iron (Fe) overload in patients with CHB [39].

In the case of chronic hepatitis C (CHC), iron (Fe) liver deposits were detected in 7–61% of patients depending on the severity of the disease [93]. Recent studies have suggested that elevated levels of iron (Fe) in the liver play a critical role in the liver disease progression as well as increasing the risk for developing liver cancer [94]. Moreover, other studies have concluded that giving iron (Fe) supplementation in the case of haemodialysis for patients who have hepatitis C virus (HCV) infection resulted in a significant increase in the levels of transaminase after only three months of therapy [4].

Mesenchymal hepatic iron (Fe) overload in patients with HCV infection was reported to result from the hepatocyte necrosis, which leads to releasing the ferritin and iron (Fe) uptake of both macrophages and Kupffer cells [95]. This was suggested to have a contribution to the release of the cytokine, which triggers liver inflammation and fibrosis [94]. Other studies have focused on the role of the hemochromatosis gene (HFE) on the cellular level and have concluded that it promotes iron (Fe) overload in patients with HCV infection [96].

Further studies have concluded that homozygous and heterozygous mutations in HFE C282Y have led to hepatic iron (Fe) overload, which promoted steatosis and fibrosis in the liver of patients with HCV infection [97]. Nonetheless, the beneficial roles of iron (Fe) on the translation of HCV in diverse HCV genotypes have been reported in recent studies; however, it is still unclear whether iron (Fe) suppresses or promotes the replication of HCV [39]. It has been clarified in other recent discussions whether iron (Fe) promotes the replication of HCV in liver cells [98], and recent studies have reported that the replication of HCV has been shown to enhance in iron (Fe) overloaded macrophages as compared to the iron (Fe) physiological level.

**Figure 3:**
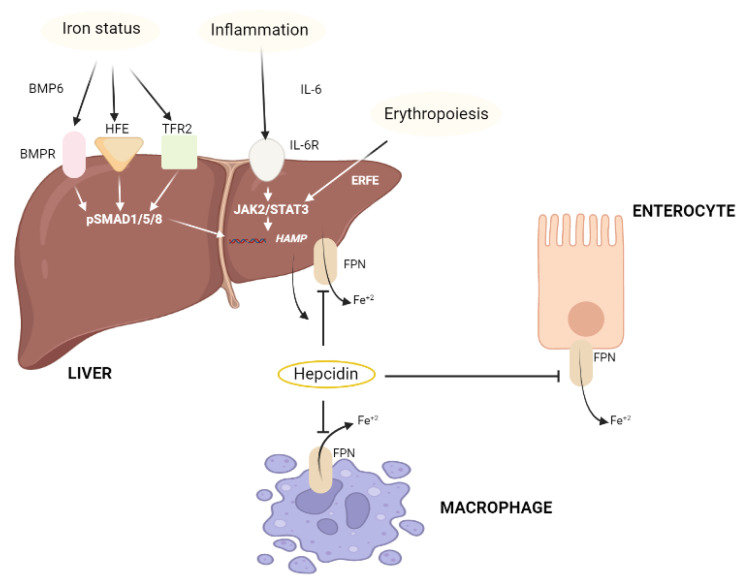
The mechanism of action and expression of hepcidin.

The results suggested that the reasons behind this might be due to the high oxidative stress in the macrophages as well as the impairment in the immune function [40]. In a study that was conducted by Fujita et al., it was reported that the hepcidin-to-ferritin ratio showed a significant decrease in patients with HCV when compared to controls or HBV patients [99]. In vitro, conflicting results have been generated concerning the antiviral role of iron (Fe). In addition, the use of human hepatocyte cell lines has revealed that iron (Fe) could either cause an enhancement or an inhibition of the HCV replication [99,100]. The mechanism of action and expression of hepcidin is illustrated in Figure 3.

#### 2.1.4. Copper (Cu)

The role that copper (Cu) plays in liver disorders can be recognized in Wilson’s disease, due to the tendency for copper (Cu) to accumulate, hence leading to the generation of cellular reactive oxygen species [101,102]. In mammalian cells, ceruloplasmin, cytochrome-c oxidase, hephaestin and copper-zinc superoxide dismutase are cuproenzymes that depend on the availability of copper (Cu) to function properly and to be metabolized [103]. A deficiency in the serum levels of copper (Cu) results in an iron (Fe) overload, cytopenia, tissue fibrosis and an increase in the susceptibility to various infections [102,103].

Acute HCV infection was shown to increase serum copper (Cu), which is then exacerbated in patients with CHC and fibrotic liver disease [6]. Hepatic copper (Cu) levels were shown to increase in patients with CHC, as it binds to MTs (Cu–MTs), hence, leading to hepatic copper (Cu) overload [4]. Cu–MTs were shown to cause a stimulation of the hydroxyl radical generation in rat models, which results in liver damage and, in some cases, fibrosis. As copper (Cu) can be solely excreted in the bile, HCV-mediated inhibition of the secretion of bile acid may cause a retention of the biliary copper (Cu) [38,104].

Remarkably, the metabolism of copper (Cu) and zinc (Zn) has been shown to take place in the liver. Studies have reported that over-supplementation of zinc (Zn) could result in a copper (Cu) deficiency due to the inhibition of the copper (Cu) absorption in the gut [4]. Excess amounts of copper (Cu) upon inflammation increases oxidative stress, and a high Cu/Zn ratio was observed in chronic inflammatory diseases, infections, as well as malnutrition [105]. It can be concluded that copper (Cu) in its different forms can show antiviral properties [106].

Particularly, cuprous oxide nanoparticles (CO-NPs) [106,107], have been shown to cause an inhibition of the HCV cell cultures infectivity at a non-cytotoxic concentration [108,109].

### 2.2. The Roles and Mechanism of Trace Elements in COVID-19

Drugs repurposing strategy has been shown to have a positive impact on the immune response and it is now widely applied in the COVID-19 pandemic [110]. The adjuvant supply of some important micronutrients that function as positive modulators in the immune system were shown to provide a further support to this strategy, in which some vitamins, such as vitamin A, B6, B12, C, D and E, as well as some essential trace elements, including zinc (Zn), iron (Fe), selenium (Se) and copper (Cu), were considered promising [25]. However, currently, the database is still very limited, and it is not yet confirmed whether some trace elements or vitamins are certainly deficient in COVID-19 patients and whether their serum concentrations are linked to the severity of the disease or to the mortality risk [48].

#### 2.2.1. Zinc (Zn)

As mentioned previously, zinc (Zn) has a role in the modulation of antiviral immunity, which affects the inflammatory response in both humans and animals. Information provided by the current clinical studies has shown that modulating Zn status may be crucial to patients with COVID-19 [47]. In coronavirus, zinc (Zn) was shown to inhibit replicase polyproteins proteolytic processing along with inhibiting the activity of RNA-dependent RNA polymerase (RdRp) [42].

A study reported that giving a high-dose intravenous zinc (HDIVZn) can provide protection to various body organs, such as the heart, liver and kidneys against hypoxic damage [43]. It was observed that elderly people are the most vulnerable group to develop severe COVID-19. Among the factors that have been considered is the weakening of the immune system that is related to the old age, which results in a low serum levels of zinc (Zn). In a recent study that was conducted on 3473 patients who have admitted to hospital with a highly severe COVID-19, confirmed that administering zinc (Zn) has a relevant role [111].

Moreover, the same study reported that the patients who were given Zn/ionophore therapy showed a 24% reduced risk of in-hospital mortality, where 17% of those patients who received Zn/ionophore survived while 12% died. Remarkably, the same study reported that patients who were given zinc (Zn) only or the ionophore alone have not shown significant improvement, which suggested that the combination of zinc (Zn) with ionophore have a synergistic action, which could be powerful for elderly patients with COVID-19 [5,41].

#### 2.2.2. Selenium (Se)

In a recent study that was conducted at the Surrey University, China, a statistically strong correlation was observed between the serum concentration of selenium (Se) and the percentages of recovery and fatality in patients with COVID-19. The results demonstrated that the provinces that have high concentrations of selenium (Se) in the soils reported a lower fatality rate from COVID-19 compared to other areas that have selenium (Se)-deficient soils [45].

Selenium (Se) deficiency in patients with COVID-19 was shown to be related to mutations, replication, as well as virulence of RNA viruses. Therefore, selenium (Se) was shown to be helpful to recover the host’s antioxidant ability, decrease in the endotheliocytes apoptosis and damage, as well as a decrease in the thrombocytes aggregation [46]. Furthermore, low selenium (Se) levels were noticed widely in patients with a higher risk of developing a severe COVID-19 infection and, in particular, in senior individuals [47].

Studies have reported that the immune system relies on a number of selenoproteins that contain selenocysteine in the active site and known to be dependent for a full expression and enzymatic activity on the abundant supply of selenium (Se). Thus, selenium (Se) deficiency is considered a risk factor for viral infections [112]. It is worth mentioning that the cure rate from COVID-19 has been recently linked to the basal selenium (Se) status in studies that have been conducted in different areas of China [45]. Together, the available published studies have shown a support to the conception that selenium (Se) may be relevant to SARS-CoV-2 infection and COVID-19 disease course [113]. However, current data on the status of selenium (Se) in individual patients who experience severe COVID-19 symptoms are missing. Thus, it is hypothesized that a severe selenium (Se) deficiency is associated with poor survival rates in COVID-19 [48].

#### 2.2.3. Copper (Cu)

Copper (Cu) plays an important role in the regular immune response. A number of cupro-enzymes were reported to have a direct effect on the general body’s developmental, metabolic and adaptive pathways [14,112]. Years after the first viral pandemic, scientists have estimated the detailed role of copper (Cu) in deactivating various viruses, including coronavirus 229E and COVID-19. William Keevil and his team recently applied copper (Cu) to fight coronavirus, where they have added copper (Cu) elements in a wide range of public places.

COVID-19 was shown to be deactivated by copper (Cu) ions that were applied to the surfaces within few hours only. The mechanism by which these ions deactivated the virus was due to the copper (Cu) ions attacking the virus’s lipid membrane, invading it and, hence destroying its nucleic acids [47]. The antiviral characteristics of copper (Cu) include inactivating the single or double-stranded DNA or RNA viruses, blocking papain-like protease 2, which is crucial for the replication of SARS-CoV-1 and destroying the viral genomes [51].

There are limited clinical data on the serum levels of copper (Cu) in COVID-19 patients. Some clinical studies have focused on pregnant women with COVID-19 and have observed a trimester-dependent increase in serum levels of copper (Cu), with only small deviations as compared to healthy control pregnancies. Remarkably, serum levels of copper (Cu) were shown to increase in pregnant women with COVID-19 particularly in the first and the third trimesters; however, serum levels of copper (Cu) have not been shown to increase in the second trimester [49].

Another recent study that was conducted in Wuhan, China indicated that copper (Cu) serum levels are generally increased in patients with severe COVID-19 infection, and no difference was observed in full blood copper (Cu) as compared to survivors and non-survivors. However, the difference in the serum concentrations of copper (Cu) related to the severity of COVID-19 was small [20]. Recently, it was reported that an increased oxidative stress and an elevation in the lipid peroxide levels were detected in patients with COVID-19 who also experience severe pneumonia and presented with a particular increase in the copper-to-zinc ratio and a drop in the levels of circulating antioxidants, such as vitamin C, selenium (Se), thiol proteins and glutathione [18]. In a study that was conducted by Lee et al., an assessment of the serum concentrations of a number of trace elements along with an evaluation of their clinical significance in a number of critically ill patients was conducted.

A decrease in copper (Cu) concentrations was seen at ICU admission. An increase in copper (Cu) levels with its substitution for the duration of the ICU stay was associated with a significant decrease in mortality as compared to lower concentration of copper (Cu) (5.6 vs. 50.0%, p ¼ 0.013) [50]. Other studies have reported that copper (Cu) supplementation has an important role in regulating IL-2, which has a critical role in the proliferation of T helper cell, the Th1 and Th2 cells balance and, the cytotoxicity of natural killer (NK) cell, which altogether have important roles in managing the immune dysregulation in critically ill COVID-19 patients [51].

A closer understanding of the signalling of copper (Cu), its vulnerability, assessment and interpretation methods, rout of administration as well as dosages are all needed to be taken into consideration regarding the therapeutic administration of copper (Cu) as part of treating critically ill COVID-19 patients. Increased attention has to be given to avoid copper (Cu) toxic limits and further work is still required to estimate the adverse effects of different copper (Cu) doses [114,115].

#### 2.2.4. Iron (Fe)

Iron (Fe) is an essential trace element that was proven to play vital roles in both eukaryotic and prokaryotic cells [116]. Several studies were conducted to assess the regulation of iron (Fe) in the defence mechanism of host cells, where they demonstrated that a decrease in the levels of iron (Fe) will lead to resistance against viral infections [117], whereas an increased level of iron (Fe) was shown to expand the virus population [118]. Recently, many studies have been conducted to analyse the status of iron in COVID-19 prognosis. In a study, it was found that the serum levels of iron (Fe) have decreased in confirmed SARS patients [119].

Other studies on COVID-19 have revealed that a decreased level of serum iron (Fe) was considered an independent risk factor for developing severe hypoxemic respiratory failure and further death in patients who had COVID-19 [120]. Moreover, an increased level of serum ferritin was found to be related to poor outcomes in COVID-19 patients [121]. A number of studies have recently demonstrated that the levels of serum ferritin in COVID-19 non-survivors have exceeded the serum ferritin levels in the survivors by two-folds.

However, it is still unclear whether hyper-ferritinemia in COVID-19 patients is a systemic marker or a modulator in disease pathogenesis. Increasing evidence has shown that oxidative stress, inflammatory conditions as well as changes in the iron (Fe) homeostasis are linked at a systemic level [122,123]. Iron (Fe) deficiency has been shown to be associated with weakened skeletal muscles and may cause a reduction in the respiratory capacity. As a result, this may worsen the COVID-19 patient’s condition and hence leads to death [124].

In a recent study that was conducted in Turkey to determine vitamin B12, vitamin D, folate and iron (Fe) levels in patients with COVID-19, it was reported that the serum levels of iron (Fe) were low. The results have also shown that that a deficiency in the serum levels of iron (Fe), vitamin D and folate and excess levels of vitamin B12, were correlated with ICU hospitalization, intubation and death [125]. Nevertheless, iron (Fe) supplementation was shown to enhance the immunity; however, it was also reported that iron can exacerbate the infection [126].

Studies that analysed SARS-CoV-1 and MERS-CoV have suggested that iron (Fe) plays a vital role in the replication of the virus [63]. In a study that was conducted by Augustine et al., it was found that oral iron (Fe) supplementation given to a patient who has an inflammatory condition might lead to oxidative stress and hence adverse gut microbiome [127]. This has raised the concerns regarding whether iron (Fe) supplementation programs are safe and effective during COVID-19 pandemic [128].

Kell et al. recently suggested that a lactoferrin supplementation could enhance the immunity, reduce inflammation and modulate the production of cytokine and ROS, which all result in a reduction in the iron (Fe) overload, due to the immunomodulatory and anti-inflammatory effects of lactoferrin and its ability to bind to different receptors especially the ones targeted by coronaviruses, hence, blocking their entry into the host cell [129].

## 3. Roles and Mechanism of Micronutrients in Diabetes Mellitus

Type-2 Diabetes Mellitus (T2DM) is affected by a mixture of both internal and external risk factors. Most studies have focused on studying the status of macronutrients as a strategy for preventing T2DM. On the other hand, micronutrients and, in particular, some trace elements (TEs) were proposed in various studies as determinants of T2DM risk [130]. Particularly, the imbalanced levels of chromium (Cr), zinc (Zn), vanadium (V), copper (Cu), selenium (Se) and iron (Fe) seem to have a role in the development and progression of T2DM [26].

Recent studies have reported that the overload or deficiency of trace elements could be related to oxidative stress, which is associated with insulin resistance and hence diabetes [131]. Additionally, chromium, zinc, copper, iron and selenium have been observed in many studies to have an antioxidant effect and might lead to an enhancement in the insulin action by activating insulin receptor sites or increasing insulin sensitivity [26]. In this section, the roles and mechanisms of two trace elements, zinc (Zn) and selenium (Se) and the electrolyte magnesium (Mg) are discussed.

### 3.1. Zinc (Zn)

Zinc (Zn) has been reported in many studies to have a distinctive role in the regulation of glycaemic control due to its antioxidant characteristics. The mechanisms by which zinc (Zn) achieves this action are the stimulation of glycolysis, decreasing gluconeogenesis and the inhibition of the activity of alpha-glucosidase in the intestine [30]. Studies that used animal models to analyse and evaluate the role of zinc (Zn) in diabetes progression have revealed that zinc (Zn) has shown a consistent role in the insulin secretion while improving the sensitivity of insulin [132]. Other studies have also reported the roles that zinc (Zn) plays to regulate the hepatic insulin clearance [133].

However, clinical studies have shown inconsistent results [134]. A study that was conducted on Finnish men reported higher serum levels of zinc (Zn) to be associated with an increased T2DM risk [52]. However, another study that was conducted on older prediabetic Australian men reported higher serum levels of zinc (Zn) to be associated with a decreased insulin resistance [53]. Other randomized controlled studies have revealed some beneficial effects of zinc (Zn) supplementation given to diabetic patients for glycemic control [30].

Pancreatic β cells were shown to have a high concentration of zinc (Zn) when compared to other cells types. Particularly, insulin secretory granules were shown to contain the highest amount of zinc (Zn) within the pancreatic β cells [54]. In T2DM, due to the antioxidative role of zinc (Zn), studies have shown that lacking zinc (Zn) could lead to the damage of pancreatic β cells following oxidative stress. In a prospective cohort study that was recently conducted in the United States, 82,000 women were analysed, and results have revealed that low zinc (Zn) intake have resulted in a 17% increase in the risk of developing diabetes when compared to other women who took sufficient amounts of zinc (Zn) [135]. In a recent study that was conducted in China, a negative correlation between plasma concentrations of zinc (Zn) and the onset of diabetes was reported [55]. Although these data have suggested that providing zinc (Zn) supplementation will prevent the glucose homeostasis disruption in zinc-deficient patients, further studies should be conducted for clarifying the role that zinc (Zn) supplementation play to prevent the onset of diabetes. On the other hand, it is worth mentioning that over-supplementation of zinc (Zn) may lead to deleterious effects, due to the unfavourable increase in the levels of HbA1c and high blood pressure [54].

### 3.2. Selenium (Se)

As mentioned in previous section, selenoproteins have antioxidant and anti-inflammatory properties [136], which suggests the beneficial effects of selenium (Se) for patients with T2DM as it is characterized by oxidative stress. Selenium (as selenate) was shown to have anti-diabetic, as well as insulin-mimetic properties, at high doses [56]. However, the observational epidemiological studies have shown that higher plasma concentration of selenium (Se) was associated with a higher T2DM occurrence [57], which leaves the role of selenium (Se) in diabetes a matter of discussion [59,122,123,124].

Many studies have conducted different assessments to figure out the relationship between the plasma levels of selenium (Se) and the common conditions that involve an increased oxidative stress and inflammatory reaction, such as T2DM [137,138,139]. A small randomized controlled trial (RCT) was conducted in the United States and showed that the administration of 200 μg/day of selenium (Se) for preventing non-melanoma skin cancer, resulted in an incidence of T2DM in patients who were reported with the highest selenium (Se) intake at baseline [140]. Several recent studies have given biological plausibility for a diabetogenic effect of selenium (Se) and selenoproteins [61]. Moreover, studies are also showing that minimized levels of environmental risk factors, such as trace elements dietary intake as well as exposure to contaminants, can affect diabetes aetiology [141].

Therefore, the chemical exposome as well as obesity, lifestyle and diabetic family history may be among the factors to be considered as additional determinants of this metabolic disease [142]. Recent experimental to epidemiologic studies have suggested a positive relationship between selenium (Se) and the higher risk of diabetes; however, the majority of these studies were conducted among prevalent cases, rendering it hard to confirm in a diabetes case whether selenium (Se) is the cause or the result [143,144].

### 3.3. Magnesium (Mg)

Magnesium (Mg) is the rate-limiting factor for a number of enzymes that are involved in the metabolism of carbohydrate and energy, as well as being essential for the intermediary metabolism to synthesize macromolecules [145]. Magnesium (Mg) deficiency has been frequently reported in obese subjects [58], and is observed in diabetic patients or in those with metabolic syndromes. Magnesium (Mg) deficiency has also been reported to increases the risk for T2DM [59]. Magnesium (Mg) depletion was shown to promote direct and indirect chronic inflammation by the modification of the intestinal microbiota [146]. T2DM has also been shown to be characterized with an altered homeostasis of magnesium (Mg), and results of the studies conducted have shown an inverse relationship between magnesium (Mg) intake and the risk of developing T2DM in a dose-response manner [60]. A recent epidemiologic study has shown a high prevalence of hypomagnesemia in type-2 diabetic subjects [61].

**Figure 4:**
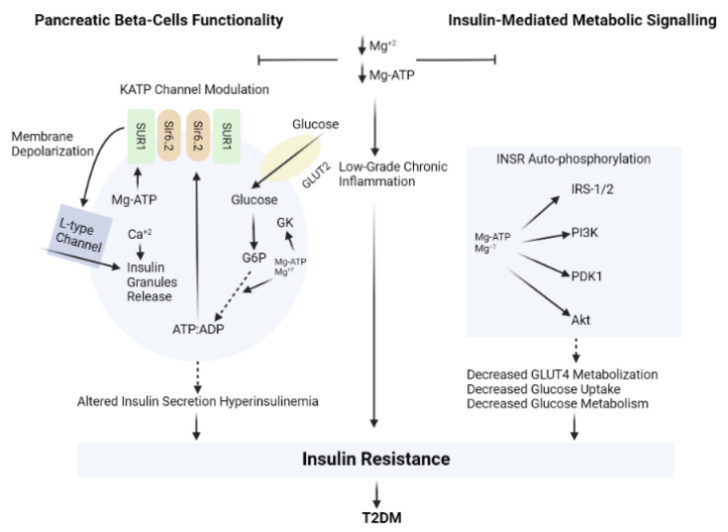
The relationship between magnesium (Mg) and insulin signalling and resistance.

Another recent study revealed that depletion of magnesium (Mg) in type-2 diabetic patients is mainly a consequence of a low magnesium (Mg) intake in addition to the increased magnesium (Mg) urinary loss, as a result of an impaired renal function [147]. Recent studies have demonstrated that hypomagnesemia was associated with the development of T2DM. The influence of magnesium (Mg) on the metabolism of glucose, insulin action and sensitivity, may give an explanation to the negative association between T2DM incidence and magnesium (Mg) intake [148,149]. The relationship between magnesium (Mg) and insulin signalling and resistance is illustrated in Figure 4.

Other studies have suggested that magnesium (Mg) deficiency could have other contributions to the progression of T2DM, such as modulating Na+/K+—ATPase, which is crucial for the maintenance of the membrane potential and low concentration of cytoplasmic sodium [150]. Studies suggested that the deficiency in magnesium (Mg) may not be a secondary consequence of T2DM; however, it may be play a role in insulin resistance and altering glucose tolerance, thus leading to the development of T2DM [151].

## 4. Current Status and Clinical Trials

Table 2 summarizes a number of clinical trials and their current status in hepatitis, covid19 and type-2 diabetes mellitus and the roles of the trace elements zinc (Zn), slelenium (Se), copper (Cu) and iron (Fe) and the electrolyte magnesium (Mg) whenever applicable [152,153,154,155,156,157,158,159,160,161,162,163,164,165,166,167,168,169,170].

## 5. Conclusions

Several studies have been conducted to assess the roles of certain vitamins and trace elements in coping with viral infections and other chronic diseases. The essential trace elements, such as copper (Cu), selenium (Se), zinc (Zn), iron (Fe) and the electrolyte magnesium (Mg), are among the most commonly studied micronutrients. Studies have reported that an impaired homeostasis of these trace elements may lead to inflammatory changes and/or metabolic abnormalities.

Zinc (Zn), selenium (Se), copper (Cu) and iron (Fe) have been studied for their involvement in the liver pathology, in particular, chronic hepatitis. Several studies have been conducted in the last decade to assess the roles and mechanisms of these trace elements in hepatic disorders. Moreover, extensive studies have focused on analysing zinc (Zn), selenium (Se) and copper (Cu) for their antiviral activities against SARS-CoV2 virus; however, the current database available remains limited, and it is not yet confirmed whether some trace elements or vitamins are certainly deficient in COVID-19 patients or whether their serum concentrations are linked to the disease severity or to the mortality risk.

Additionally, Cr, Zn, Cu, Fe and Se have been observed in many studies to have an antioxidant effect and might lead to enhancement in the insulin action by activating insulin receptor sites or increasing insulin sensitivity. However, it is worth mentioning that over-supplementation of some of these trace elements, for instance zinc (Zn), may lead to deleterious effects due to the unfavourable increase in the levels of HbA1c and high blood pressure. Recent advances in newly discovered molecular biological techniques have facilitated the discovery of the novel mechanisms by which an impairment in the metabolism of these trace elements causes different metabolic abnormalities. Further studies are still required for assessment of the dosing and toxicity of these trace elements.

## Figures and Tables

**Table 1:** Pathogenic pathways and roles of a number of micronutrients in the prevention and/or treatment of chronic hepatitis, COVID-19 and type-2 diabetes mellitus.

	Chronic Hepatitis
**Zinc (Zn)**	The two human zinc finger antiviral protein (hZAP) isoforms (i.e., h-ZAP S; h-ZAP L) inhibited the replication of HBV in human hepatocyte-derived cells [31]. Knock-down of the expression of ZAP increased the HBV RNA levels and caused a partial attenuation of the antiviral effect stimulated by IPS-1 in the cell cultures used [32]. Zinc (Zn) serum levels were shown to rise significantly upon viral eradication using interferon (IFN)-based regimens or direct-acting anti-viral (DAA) therapy [33]. Zinc (Zn) treatment was shown to significantly inhibit the replication of the virus in the human hepatoma cell culture of genotype-1 and genotype-3 HEV by inhibiting the RNA-dependent RNA polymerase (RdRp) viral activity in vitro [7]. A decline in the serum levels of zinc following an IFN-α treatment resulted in an increase in the baseline zinc (Zn) levels, causing a stimulation of the metallothionein (MT) expression and the anti-viral activity [34].
**Selenium (Se)**	CHC infection reduced the serum levels of selenium (Se) and the activity of glutathion peroxidase (GPx) [35,36].
**Iron (Fe)**	An enhancement in the progression of chronic HBV infection was related to the high iron (Fe) serum levels [37]. The levels of serum iron (Fe) and serum ferritin were increased in patients with chronic hepatitis B (CHB) [38]. Some inflammatory factors, such as liver injury, micro ribonucleic acid -122, viral activity, ROS, IL-6 could result in an iron (Fe) overload in patients with CHB [39]. The replication of HCV was shown to enhance in iron (Fe) overloaded macrophages as compared to the iron (Fe) physiological level due to the high oxidative stress in the macrophages and the impairment in the immune function [40].
**Copper (Cu)**	Acute HCV infection increased serum copper (Cu) as it binds to MTs (Cu–MTs), hence leading to hepatic copper overload [6]. HCV-mediated inhibition of the secretion of bile acid may cause a retention of the biliary copper(Cu) [41]. Over-supplementation of zinc (Zn) could result in a copper (Cu) deficiency due to the inhibition of the copper absorption in the gut [4]. Cuprous oxide nanoparticles (CO-NPs) inhibit the HCV cell cultures infectivity at a non-cytotoxic concentration [5].
	**COVID-19**
**Zinc (Zn)**	Zinc (Zn) inhibits replicase polyproteins proteolytic processing and inhibits the activity of RNA-dependent RNA polymerase (RdRp) [42]. Giving a high-dose intravenous zinc (HDIVZn) provides a protection to various body organs, such as the heart, liver and kidneys against the hypoxic damage [43]. The combination of zinc (Zn) with ionophore have a synergistic action, which could be powerful for elderly patients with COVID-19 [44].
**Selenium (Se)**	The provinces that have high concentrations of selenium (Se) in the soils showed lower fatality from COVID-19 as compared to other areas with selenium (Se) deficient soils [45]. Selenium (Se) deficiency in patients with COVID-19 was shown to be related to mutations, replication, as well as virulence of RNA viruses [46]. Low selenium (Se) levels were noticed widely in patients with a higher risk of developing a severe COVID-19 infection and, in particular in senior individuals [47]. Severe selenium (Se) deficiency is associated with poor survival rates in COVID-19 [48].
**Copper (Cu)**	Serum levels of copper (Cu) were shown to increase in pregnant women with COVID-19 particularly in the first and the third trimesters [49]. Copper (Cu) serum levels are generally increased in patients with severe COVID-19 infection [20]. A decrease in copper (Cu) concentrations was seen at ICU admission [50]. Copper (Cu) supplementation has an important role in regulating IL-2 and plays an important role in managing the immune dysregulation in critically ill COVID-19 patients [51].
	**Type-2 Diabetes Mellitus (T2DM)**
**Zinc (Zn)**	Zinc (Zn) has a distinctive role in the regulation of glycemic control due to its antioxidant characteristics. Zinc (Zn) stimulates glycolysis, decreases gluconeogenesis and inhibits the activity of alpha-glucosidase in the intestine [30]. Higher serum levels of zinc (Zn) are associated with an increased T2DM risk [52]. Higher serum levels of zinc (Zn) are associated with a decreased insulin resistance [53]. Pancreatic β cells have a high concentration of zinc (Zn) when compared to other cells types [54]. A negative correlation between plasma concentrations of zinc (Zn) and the onset of diabetes was reported [55]. Over-supplementation of zinc (Zn) may increase the levels of HbA1c and blood pressure [55].
**Selenium (Se)**	Selenium (as selenate) has anti-diabetic, as well as insulin-mimetic properties, at high doses [56]. Higher plasma concentration of selenium (Se) was associated with a higher T2DM occurrence [57].
**Magnesium (Mg)**	Magnesium (Mg) deficiency has been frequently reported in obese subjects and is observed in diabetic patients or in those with metabolic syndromes [58]. Magnesium (Mg) deficiency increases the risk for T2DM [59]. T2DM is characterized with an altered homeostasis of magnesium (Mg) [60]. A high prevalence of hypomagnesemia in type-2 diabetic subjects was detected [61].

**Table 2:** Database of clinical trials on zinc (Zn), selenium (Se), copper (Cu), magnesium (Mg) and iron (Fe) interventions against hepatitis, covid19 and type-2 diabetes mellitus.

ClinicalTials.gov Identifier	Study Title	Condition	Intervention & Findings	Status	Reference
**NCT05000762**	Zinc Supplementation Improves Cardiovascular Morbidity in Patients with Diabetes Mellitus	Diabetes Mellitus, Type 2 Cardiovascular Diseases	Dietary Supplement: Zinc (Zn) Zinc gluconate 30 mg/day orally	Recruiting	[152]
**NCT05320510**	Effect of Selenium (Se) Supplementation on Glycemic Control in Patients with Type 2 Diabetes or Prediabetes	Type 2 Diabetes Pre Diabetes	Dietary Supplement: Se-yeast The participants will be asked to take Se-yeast tablet. Dietary Supplement: Placebo The participants will be asked to take placebo-yeast tablet.	Not yet recruiting	[153]
**NCT04636411**	Effect of Oral Magnesium Supplementation on Patients with Type 2 Diabetes	Type2 Diabetes	Dietary Supplement: Oral Magnesium (Mg) Supplementation Other: Standard Care for diabetic patients	Recruiting	[154]
**NCT03002545**	Magnesium Supplementation in Type II Diabetes	Effect of magnesium (Mg) in diabetes	Dietary Supplement: Magnesium (Mg) citrate Dietary Supplement: Placebo Findings: oral Mg citrate supplementation reduced HbA1c levels and reduced BP in normomagnesemic persons with MetS	Completed	[155]
**NCT05033054**	Effect of Dietary Magnesium Supplementation vs. Dapagliflozin in Patients with Diabetic Kidney Disease (DKD)	Kidney Disease, Chronic Diabetes	Dietary Supplement: EffCaMg Citrate 480 mg Drug: Dapagliflozin 10 mg Dietary Supplement: Placebo EffCaMg Citrate Drug: Placebo Dapagliflozin	Not yet recruiting	[156]
**NCT04869579**	Selenium (Se) as a Potential Treatment for Moderately-ill, Severely-ill and Critically-ill COVID-19 Patients.	Covid19	Drug: Selenium (as Selenious Acid)Other: Placebo	Not yet recruiting	[157]
**NCT04877509**	Micronutrient Status Involved in Immunity in Elderly Patients with COVID-19	Covid19	Biological: Selenium (Se), Zinc (Zn) and Copper (Cu), Vitamin A, D, E plasma concentrations during patient hospitalization	Completed	[158]
**NCT04941703**	CHANGE COVID-19 Severity	COVID-19 Infection	Drug: Magnesium Citrate plus probiotic	Recruiting	[159]
**NCT04716985**	Evaluation of the Daily Intake of 0.5 L of Water Saturated with Molecular Hydrogen for 21 Days in COVID-19 Patients Treated in Ambulatory Care	SARS-CoV-2 Covid19 AMBULATORY CARE	Dietary Supplement: molecular hydrogen Dietary Supplement: placebo magnesium (Mg)	Active not recruiting	[160]
**NCT04641195**	Vitamin D and Zinc Supplementation for Improving Treatment Outcomes Among COVID-19 Patients in India	Covid19	Dietary Supplement: Vitamin D3 (cholecalciferol) Dietary Supplement: Zinc (zinc gluconate) Dietary Supplement: Zinc (zinc gluconate) & Vitamin D (cholecalciferol) Other: Placebo	recruiting	[161]
**NCT04370782**	Hydroxychloroquine and Zinc with Either Azithromycin or Doxycycline for Treatment of COVID-19 in Outpatient Setting	Covid19	Drug: Hydroxychloroquine Drug: Azithromycin Drug: Zinc Sulfate Drug: Doxycycline	Completed	[162]
**NCT04558424**	RCT, Double Blind, Placebo to Evaluate the Effect of Zinc and Ascorbic Acid Supplementation in COVID-19 Positive Hospitalized Patients in BSMMU	Covid19	Dietary Supplement: zinc gluconate and ascorbic acid	Not yet recruiting	[163]
**NCT04542993**	Can SARS-CoV-2 Viral Load and COVID-19 Disease Severity Be Reduced by Resveratrol-assisted Zinc Therapy (Reszinate)	Covid19 SARS-CoV Infection	Dietary Supplement: Zinc Picolinate Dietary Supplement: Resveratrol Dietary Supplement: Zinc Picolinate Placebo Dietary Supplement: Resveratrol Placebo	Active, not recruiting	[164]
**NCT04072822**	Trial of Anakinra (Plus Zinc) or Prednisone in Patients with Severe Alcoholic Hepatitis	Alcoholic Hepatitis	Drug: Anakinra and Zinc (Zn) Drug: Prednisone Drug: Placebos	Active, not recruiting	[165]
**NCT01809132**	Efficacy Study of Anakinra, Pentoxifylline and Zinc Compared to Methylprednisolone in Severe Acute Alcoholic Hepatitis	Acute Alcoholic Hepatitis	Drug: Anakinra Drug: Pentoxifylline Drug: Zinc Sulfate Drug: Methylprednisolone	Completed, has results	[166]
**NCT01355107**	Comparison of Selenium (Se) Levels in HCV- Infected Patients at Different Stages of Disease	Hepatitis C Liver Cirrhosis Carcinoma, Hepatocellular	N/A	Completed	[167]
**NCT03349008**	Magnesium Isoglycyrrhizinate Followed by Diammonium Glycyrrhizinate and Combined with Entecavir in Chronic Hepatitis B	Chronic Hepatitis B Liver Inflammation	Drug: Entecavir Drug: Magnesium Isoglycyrrhizinate Drug: Diammonium Glycyrrhizinate Drug: Magnesium Isoglycyrrhizinate Placebo Drug: Diammonium Glycyrrhizinate Placebo	N/A	[168]
**NCT03166280**	Hepatitis c and Vitamin D and Iron (Fe) Status	Hepatitis C	Drug: Sofosbuvir 400 mg Drug: Daclatasvir 60 mg/day	N/A	[169]
**NCT02744560**	Effect of Spirulina on Liver Iron (Fe) Concentration in Beta Thalassemic Children with Hepatitis C	Beta Thalassemia Major	Dietary Supplement: spirulina	Completed	[170]

## Data Availability

Not applicable.

## Abbreviations

CHC	Chronic hepatitis C
CO-NPs	Coprous oxide nanoparticles
COVID	Coronavirus disease
Cu	Copper
DAA	Direct acting antiviral
DPP	Diabetes prevention program
Fe	Iron
GPX1	Glutathione peroxidase-1
H1N1	Human influenza
HBV	Hepatitis B Virus
HCV	Hepatitis C Virus
HDIVZn	High dose intravenous zinc
HEV	Hepatitis E Virus
Huh	Human hepatoma
hZAP	Human zinc finger antiviral protein
ICU	Intensive care unit
IFN	Interferon
IL	Interleukin
Mg	Magnesium
mRNA	Messenger ribonucleic acid
MT	Metallothionein
NK cell	Natural killer cell
pgRNA	Pregenomic ribonucleic acid
RCT	Randomized controlled trial
RdRp	RNA-dependent RNA polymerase
ROS	Reactive oxygen species
Se	Selenium
SELENOP	Selenoproteins
T2DM	Type-2 diabetes mellitus
TEs	Trace elements
TH cell	T helper cell
TXNRD	Thioredoxin reductase
Zn	Zinc

## References

[1] Bhattacharya P.T., Misra S.R., Hussain M. (2016). Nutritional Aspects of Essential Trace Elements in Oral Health and Disease: An Extensive Review. Scientifica.

[2] Attar T. (2020). A Mini-Review on Importance and Role of Trace Elements in the Human Organism. Chem. Rev. Lett..

[3] Şahin M., Karayakar F., Erdogan K.E., Bas F., Colak T. (2018). Liver Tissue Trace Element Levels in HepB Patients and the Relationship of These Elements with Histological Injury in the Liver and with Clinical Parameters. J. Trace Elem. Med. Biol..

[4] Gupta S., Read S.A., Shackel N.A., Hebbard L., George J., Ahlenstiel G. (2019). The Role of Micronutrients in the Infection and Subsequent Response to Hepatitis C Virus. Cells.

[5] Hang X., Peng H., Song H., Qi Z., Miao X., Xu W. (2015). Antiviral Activity of Cuprous Oxide Nanoparticles against Hepatitis C Virus in Vitro. J. Virol. Methods.

[6] Guo C.H., Chen P.C., Ko W.S. (2013). Status of Essential Trace Minerals and Oxidative Stress in Viral Hepatitis C Patients with Nonalcoholic Fatty Liver Disease. Int. J. Med. Sci..

[7] Kaushik N., Subramani C., Anang S., Muthumohan R., Shalimar Nayak B., Ranjith-Kumar C.T., Surjit M. (2017). Crossm Zinc Salts Block Hepatitis E Virus. J. Virol..

[8] Kumar A., Kubota Y., Chernov M., Kasuya H. (2020). Potential role of zinc supplementation in prophylaxis and treatment of COVID-19. Med. Hypotheses.

[9] Chasapis C.T., Georgiopoulou A.K., Perlepes S.P., Bjørklund G., Peana M. (2021). A SARS-CoV-2 -human metalloproteome interaction map. J. Inorg. Biochem..

[10] Andreou A., Trantza S., Filippou D., Filippou D., Sipsas N., Tsiodras S. (2020). COVID-19: The Potential Role of Copper and N-Acetylcysteine (NAC) in a Combination of Candidate Antiviral Treatments against SARS-CoV-2. In Vivo (Brooklyn).

[11] Gattermann N., Muckenthaler M.U., Kulozik A.E., Metzgeroth G., Hastka J. (2021). Investigation of Iron Deficiency and Iron Overload. Dtsch. Arztebl. Int..

[12] Ivanova I.D., Pal A., Simonelli I., Atanasova B., Ventriglia M., Rongioletti M., Squitti R. (2022). Evaluation of Zinc, Copper, and Cu:Zn Ratio in Serum, and Their Implications in the Course of COVID-19. J. Trace Elem. Med. Biol..

[13] Hackler J., Heller R.A., Sun Q., Schwarzer M., Diegmann J., Bachmann M., Moghaddam A., Schomburg L. (2021). Relation of Serum Copper Status to Survival in COVID-19. Nutrients.

[14] Asprouli E., Kalafati I.P., Sakellari A., Karavoltsos S., Vlachogiannakos J., Revenas K., Kokkinos A., Dassenakis M., Dedoussis G.V., Kalogeropoulos N. (2019). Evaluation of Plasma Trace Elements in Different Stages of Nonalcoholic Fatty Liver Disease. Biol. Trace Elem. Res..

[15] Parlakgül G., Arruda A.P., Pang S., Cagampan E., Min N., Güney E., Lee G.Y., Inouye K., Hess H.F., Xu C.S. (2022). Regulation of Liver Subcellular Architecture Controls Metabolic Homeostasis. Nature.

[16] Kozeniecki M., Ludke R., Kerner J., Patterson B. (2020). Micronutrients in Liver Disease: Roles, Risk Factors for Deficiency, and Recommendations for Supplementation. Nutr. Clin. Pract..

[17] Zhu L.C., Chen X.J., Kong X., Cai Y.D. (2016). Investigation of the Roles of Trace Elements during Hepatitis C Virus Infection Using Protein-Protein Interactions and a Shortest Path Algorithm. Biochim. Biophys. Acta—Gen. Subj..

[18] Pincemail J., Cavalier E., Charlier C., Cheramy–bien J.P., Brevers E., Courtois A., Fadeur M., Meziane S., Goff C.L., Misset B. (2021). Oxidative Stress Status in COVID-19 Patients Hospitalized in Intensive Care Unit for Severe Pneumonia. A Pilot Study. Antioxidants.

[19] Nedić O., Šunderić M., Robajac D., Miljuš G., Četić D., Penezić A. (2022). Major Trace Elements and Their Binding Proteins in the Early Phase of COVID-19 Infection. J. Biol. Inorg. Chem..

[20] Zeng H.L., Yang Q., Yuan P., Wang X., Cheng L. (2021). Associations of Essential and Toxic Metals/Metalloids in Whole Blood with Both Disease Severity and Mortality in Patients with COVID-19. FASEB J..

[21] De Jesus J.R., De Araújo Andrade T. (2020). Understanding the Relationship between Viral Infections and Trace Elements from a Metallomics Perspective: Implications for COVID-19. Metallomics.

[22] Fooladi S., Matin S., Mahmoodpoor A. (2020). Copper as a Potential Adjunct Therapy for Critically Ill COVID-19 Patients. Clin. Nutr. ESPEN.

[23] Nedjimi B. (2021). Can Trace Element Supplementations (Cu, Se, and Zn) Enhance Human Immunity against COVID-19 and Its New Variants?. Beni-Suef Univ. J. Basic Appl. Sci..

[24] Taheri M., Bahrami A., Habibi P., Nouri F. (2021). A Review on the Serum Electrolytes and Trace Elements Role in the Pathophysiology of COVID-19. Biol. Trace Elem. Res..

[25] Calder P.C., Carr A.C., Gombart A.F., Eggersdorfer M. (2020). Reply to “Comment on: Optimal Nutritional Status for a Well-Functioning Immune System Is an Important Factor to Protect against Viral Infections. *Nutrients*
**2020**, *12*, 1181”. Nutrients.

[26] Rodríguez-Pérez C., Gómez-Peña C., Pérez-Carrascosa F.M., Vrhovnik P., Echeverría R., Salcedo-Bellido I., Mustieles V., Željka F., Arrebola J.P. (2021). Trace Elements Concentration in Adipose Tissue and the Risk of Incident Type 2 Diabetes in a Prospective Adult Cohort. Environ. Pollut..

[27] Himoto T., Masaki T. (2020). Current Trends of Essential Trace Elements in Patients with Chronic Liver Diseases. Nutrients.

[28] Siddiqui K., Bawazeer N., Scaria Joy S. (2014). Variation in Macro and Trace Elements in Progression of Type 2 Diabetes. Sci. World J..

[29] The National Diabetes Prevention Program (NDPP) (2002). The Diabetes Prevention Program (DPP). Diabetes Care.

[30] Kant R., Verma V., Patel S., Chandra R., Chaudhary R., Shuldiner A.R., Munir K.M. (2021). Effect of Serum Zinc and Copper Levels on Insulin Secretion, Insulin Resistance and Pancreatic β Cell Dysfunction in US Adults: Findings from the National Health and Nutrition Examination Survey (NHANES) 2011–2012. Diabetes Res. Clin. Pract..

[31] Yousaf T., Sun Y., Naz W., Liu Y., Xu J., Yuan S., Wu K., Wang M., Wang J., Guo M. (2022). Multiomics Analysis of Endocytosis upon HBV Infection and Identification of SCAMP1 as a Novel Host Restriction Factor against HBV Replication. Int. J. Mol. Sci..

[32] Mao R., Nie H., Cai D., Zhang J., Liu H., Yan R., Cuconati A., Block T.M., Guo J.T., Guo H. (2013). Inhibition of Hepatitis B Virus Replication by the Host Zinc Finger Antiviral Protein. PLoS Pathog..

[33] Kuwano A., Yada M., Nagasawa S., Tanaka K., Morita Y., Masumoto A., Motomura K. (2022). Serum α-Fetoprotein Level at Treatment Completion Is a Useful Predictor of Hepatocellular Carcinoma Occurrence More than One Year after Hepatitis C Virus Eradication by Direct-Acting Antiviral Treatment. J. Viral Hepat..

[34] Dai H., Wang L., Li L., Huang Z., Ye L. (2021). Metallothionein 1: A New Spotlight on Inflammatory Diseases. Front. Immunol..

[35] Xq W., Cm L., Chen L., Ck C., Zf Y., Yx C. (2013). Selenium Levels in Patients with Hepatitis C Virus-Related Chronic Hepatitis, Liver Cirrhosis, and Hepatocellular Carcinoma: A Pilot Study. Hepatology.

[36] Petrović S., Maletić M., Lakić N., Aleksić N., Maletić J., Ristanić M., Stanimirović Z. (2021). The Effects of Antioxidants Provided with Feed on Certain Quality Parameters of Bull Semen under Heat Stress Conditions. Acta Vet..

[37] Wei Y., Ye W., Zhao W. (2018). Serum Iron Levels Decreased in Patients with HBV-Related Hepatocellular Carcinoma, as a Risk Factor for the Prognosis of HBV-Related HCC. Front. Physiol..

[38] Gao Y.H., Wang J.Y., Liu P.Y., Sun J., Wang X.M., Wu R.H., He X.T., Tu Z.K., Wang C.G., Xu H.Q. (2018). Iron Metabolism Disorders in Patients with Hepatitis B-Related Liver Diseases. World J. Clin. Cases.

[39] Yang Y.M., Cho Y.E., Hwang S. (2022). Crosstalk between Oxidative Stress and Inflammatory Liver Injury in the Pathogenesis of Alcoholic Liver Disease. Int. J. Mol. Sci..

[40] Cao X.L., Zhao M.F., Li D.G., Xing Y., Zhang Y.C., Chen J., He X.Y., Cui R., Meng J.X., Xiao X. (2016). Establishment of Macrophage Model of Iron Overload in Vitro and the Injury Induced by Oxidative Stress on Macrophage with Iron Overload. Zhonghua Yi Xue Za Zhi.

[41] Tao T.Y., Gitlin J.D. (2003). Hepatic Copper Metabolism: Insights from Genetic Disease. Hepatology.

[42] Read S.A., Obeid S., Ahlenstiel C., Ahlenstiel G. (2019). The Role of Zinc in Antiviral Immunity. Adv Nutr..

[43] Cheung E., Nikfarjam M., Jackett L., Bolton D.M., Ischia J., Patel O. (2020). The Protective Effect of Zinc Against Liver Ischaemia Reperfusion Injury in a Rat Model of Global Ischaemia. J. Clin. Exp. Hepatol..

[44] Frontera J.A., Rahimian J.O., Yaghi S., Liu M., Lewis A., De Havenon A., Mainali S., Huang J., Scher E., Wisniewski T. (2020). Treatment with Zinc is Associated with Reduced In-Hospital Mortality Among COVID-19 Patients: A Multi-Center Cohort Study. Res. Sq..

[45] Zhang J., Taylor E.W., Bennett K., Saad R., Rayman M.P. (2020). Association between Regional Selenium Status and Reported Outcome of COVID-19 Cases in China. Am. J. Clin. Nutr..

[46] Hiffler L., Rakotoambinina B. (2020). Selenium and RNA Virus Interactions: Potential Implications for SARS-CoV-2 Infection (COVID-19). Front. Nutr..

[47] Ermakov V.V., Jovanović L.N. (2022). Biological Role of Trace Elements and Viral Pathologies. Geochem. Int..

[48] Moghaddam A., Heller R.A., Sun Q., Seelig J., Cherkezov A., Seibert L., Hackler J., Seemann P., Diegmann J., Pilz M. (2020). Selenium Deficiency Is Associated with Mortality Risk from COVID-19. Nutrients.

[49] Anuk A.T., Polat N., Akdas S., Erol S.A., Tanacan A., Biriken D., Keskin H.L., Moraloglu Tekin O., Yazihan N., Sahin D. (2021). The Relation Between Trace Element Status (Zinc, Copper, Magnesium) and Clinical Outcomes in COVID-19 Infection During Pregnancy. Biol. Trace Elem. Res..

[50] Lee Y.H., Bang E.S., Lee J.H., Lee J.D., Kang D.R., Hong J., Lee J.M. (2019). Serum Concentrations of Trace Elements Zinc, Copper, Selenium, and Manganese in Critically Ill Patients. Biol. Trace Elem. Res..

[51] Raha S., Mallick R., Basak S., Duttaroy A.K. (2020). Is Copper Beneficial for COVID-19 Patients?. Med. Hypotheses.

[52] Yary T., Virtanen J.K., Ruusunen A., Tuomainen T.P., Voutilainen S. (2016). Serum Zinc and Risk of Type 2 Diabetes Incidence in Men: The Kuopio Ischaemic Heart Disease Risk Factor Study. J. Trace Elem. Med. Biol..

[53] Vashum K.P., McEvoy M., Milton A.H., Islam M.R., Hancock S., Attia J. (2014). Is Serum Zinc Associated with Pancreatic Beta Cell Function and Insulin Sensitivity in Pre-Diabetic and Normal Individuals? Findings from the Hunter Community Study. PLoS ONE.

[54] Fukunaka A., Fujitani Y. (2018). Role of Zinc Homeostasis in the Pathogenesis of Diabetes and Obesity. Int. J. Mol. Sci..

[55] Shan Z., Bao W., Zhang Y., Rong Y., Wang X., Jin Y., Song Y., Yao P., Sun C., Hu F.B. (2014). Interactions between Zinc Transporter-8 Gene (SLC30A8) and Plasma Zinc Concentrations for Impaired Glucose Regulation and Type 2 Diabetes. Diabetes.

[56] Steinbrenner H. (2013). Interference of Selenium and Selenoproteins with the Insulin-Regulated Carbohydrate and Lipid Metabolism. Free Radic. Biol. Med..

[57] Rayman M.P., Stranges S. (2013). Epidemiology of Selenium and Type 2 Diabetes: Can We Make Sense of It?. Free Radic. Biol. Med..

[58] De Baaij J.H.F., Hoenderop J.G.J., Bindels R.J.M. (2015). Magnesium in Man: Implications for Health and Disease. Physiol. Rev..

[59] Von Ehrlich B., Barbagallo M., Classen H.G., Guerrero-Romero F., Mooren F.C., Rodriguez-Moran M., Vierling W., Vormann J., Kisters K. (2017). Significance of Magnesium in Insulin Resistance, Metabolic Syndrome, and Diabetes—Recommendations of the Association of Magnesium Research e.V. Trace Elem. Electrolytes.

[60] Bertinato J., Wang K.C., Hayward S. (2017). Serum Magnesium Concentrations in the Canadian Population and Associations with Diabetes, Glycemic Regulation, and Insulin Resistance. Nutrients.

[61] Lee Y.S., Olefsky J. (2021). Chronic Tissue Inflammation and Metabolic Disease. Genes Dev..

[62] Grubaugh N.D., Ladner J.T., Lemey P., Pybus O.G., Rambaut A., Holmes E.C., Andersen K.G. (2019). Tracking Virus Outbreaks in the Twenty-First Century. Nat. Microbiol..

[63] Liu W., Zhang S., Nekhai S., Liu S. (2020). Depriving Iron Supply to the Virus Represents a Promising Adjuvant Therapeutic Against Viral Survival. Curr. Clin. Microbiol. Rep..

[64] Misumi I., Mitchell J.E., Lund M.M., Cullen J.M., Lemon S.M., Whitmire J.K. (2021). T Cells Protect against Hepatitis A Virus Infection and Limit Infection-Induced Liver Injury. J. Hepatol..

[65] Sahin M., Karayakar F., Koksal A.R., Yetim A., İyisoy M.S., Şen İ., Alkım H., Alkım C., Colak T. (2019). Changes in Liver Tissue Trace Element Concentrations During Hepatitis B Viral Infection Treatment. Biol. Trace Elem. Res..

[66] Nangliya V., Sharma A., Yadav D., Sunder S., Nijhawan S., Mishra S. (2015). Study of Trace Elements in Liver Cirrhosis Patients and Their Role in Prognosis of Disease. Biol. Trace Elem. Res..

[67] El-Megharbel S.M., Al-Thubaiti E.H., Safa H., Qahl R.A.A.-E., Reham Z.H. (2022). Synthesis and Spectroscopic Characterization of Dapagliflozin/Zn (II), Cr (III) and Se (IV) Novel Complexes That Ameliorate Hepatic Damage, Hyperglycemia and Oxidative Injury Induced by Streptozotocin-Induced Diabetic Male Rats and Their Antibacterial Act. Crystals.

[68] Lin Y., He F., Lian S., Xie B., Liu T., He J., Liu C. (2022). Selenium Status in Patients with Chronic Liver Disease: A Systematic Review and Meta-Analysis. Nutrients.

[69] Diglio D.C., Fernandes S.A., Stein J., Azeredo-da-Silva A., De Mattos A.A., Tovo C.V. (2020). Role of Zinc Supplementation in the Management of Chronic Liver Diseases: A Systematic Review and Meta-Analysis. Ann. Hepatol..

[70] Coni P., Pichiri G., Lachowicz J.I., Ravarino A., Ledda F., Fanni D., Gerosa C., Piras M., Coghe F., Gibo Y. (2021). Zinc as a Drug for Wilson’s Disease, Non-Alcoholic Liver Disease and COVID-19-Related Liver Injury. Molecules.

[71] Miwa T., Hanai T., Toshihide M., Ogiso Y., Imai K., Suetsugu A., Takai K., Shiraki M., Katsumura N., Shimizu M. (2021). Zinc Deficiency Predicts Overt Hepatic Encephalopathy and Mortality in Liver Cirrhosis Patients with Minimal Hepatic Encephalopathy. Hepatol. Res..

[72] Cunha T.A., Vermeulen-Serpa K.M., Grilo E.C., Leite-Lais L., Brandão-Neto J., Vale S.H.L. (2022). Association between Zinc and Body Composition: An Integrative Review. J. Trace Elem. Med. Biol..

[73] Barbara M., Mindikoglu A.L. (2021). The Role of Zinc in the Prevention and Treatment of Nonalcoholic Fatty Liver Disease. Metab. Open.

[74] Grüngreiff K., Reinhold D., Wedemeyer H. (2016). The Role of Zinc in Liver Cirrhosis. Ann. Hepatol..

[75] Girirajan S., Campbell C., Eichler E. (2011). EASL Clinical Practice Guidelines on Nutrition in Chronic Liver Disease. Physiol. Behav..

[76] Guo H., Jiang D., Ma D., Chang J., Dougherty A.M., Cuconati A., Block T.M., Guo J.-T. (2009). Activation of Pattern Recognition Receptor-Mediated Innate Immunity Inhibits the Replication of Hepatitis B Virus in Human Hepatocyte-Derived Cells. J. Virol..

[77] Uprichard S.L., Wieland S.F., Althage A., Chisari F.V. (2003). Transcriptional and Posttranscriptional Control of Hepatitis B Virus Gene Expression. Proc. Natl. Acad. Sci. USA.

[78] Kmiec D., Lista-Brotos M.-J., Ficarelli M., Swanson C.M., Neil S.J. (2021). The C-Terminal PARP Domain of the Long ZAP Isoform Contributes Essential Effector Functions for CpG-Directed Antiviral Activity. bioRxiv.

[79] Ficarelli M., Neil S.J.D., Swanson C.M. (2021). Targeted Restriction of Viral Gene Expression and Replication by the ZAP Antiviral System. Annu. Rev. Virol..

[80] Palomo ig Palomo I.G., Jaramillo J.C., Alarcon M.L., Gutierrez C.L., Moore-Carrasco R., Segovia F.M., Leiva E.M., Mujica V.E., Icaza G., Diaz N.S. (2008). Increased Concentrations of Soluble Vascular Cell Adhesion Molecule-1 and Soluble CD40L in Subjects with Metabolic Syndrome. Molecular. Mol. Med. Rep..

[81] Shen J., Qi W., Dai J., Leng S., Jiang K., Zhang Y., Ran S., Li C., Wen T. (2022). Tenofovir vs. Entecavir on Recurrence of Hepatitis B Virus-Related Hepatocellular Carcinoma beyond Milan Criteria after Hepatectomy. Chin. Med. J..

[82] Read S.A., O’Connor K.S., Suppiah V., Ahlenstiel C.L.E., Obeid S., Cook K.M., Cunningham A., Douglas M.W., Hogg P.J., Booth D. (2017). Zinc Is a Potent and Specific Inhibitor of IFN-Λ3 Signalling. Nat. Commun..

[83] Kaushik N., Anang S., Ganti K.P., Surjit M. (2018). Zinc: A Potential Antiviral Against Hepatitis e Virus Infection?. DNA Cell Biol..

[84] Tang C., Li S., Zhang K., Li J., Han Y., Zhan T., Zhao Q., Guo X., Zhang J. (2020). Selenium Deficiency-Induced Redox Imbalance Leads to Metabolic Reprogramming and Inflammation in the Liver. Redox Biol..

[85] Regina B.F., Gladyshev V.N., Arnér E.S., Berry M.J., Bruford E.A., Burk R.F., Carlson B.A., Castellano S., Chavatte L., Conrad M. (2016). Selenoprotein Gene Nomenclature. J. Biol. Chem..

[86] Guo Z., Chen W., Dai G., Huang Y. (2020). Cordycepin Suppresses the Migration and Invasion of Human Liver Cancer Cells by Downregulating the Expression of CXCR4. Int. J. Mol. Med..

[87] Reja M., Makar M., Visaria A., Marino D., Rustgi V. (2020). Increased Serum Selenium Levels Are Associated with Reduced Risk of Advanced Liver Fibrosis and All-Cause Mortality in NAFLD Patients: National Health and Nutrition Examination Survey (NHANES) III. Ann. Hepatol..

[88] Ko E., Kim J.S., Ju S., Seo H.W., Chang Y., Kang J.A., Park S.G., Jung G. (2018). Oxidatively Modified Protein-Disulfide Isomerase–Associated 3 Promotes Dyskerin Pseudouridine Synthase 1–Mediated Malignancy and Survival of Hepatocellular Carcinoma Cells. Hepatology.

[89] Lesnichaya M., Karpova E., Sukhov B. (2021). Effect of High Dose of Selenium Nanoparticles on Antioxidant System and Biochemical Profile of Rats in Correction of Carbon Tetrachloride-Induced Toxic Damage of Liver. Colloids Surf. B Biointerfaces.

[90] Morbitzer M., Herget T. (2005). Expression of Gastrointestinal Glutathione Peroxidase Is Inversely Correlated to the Presence of Hepatitis C Virus Subgenomic RNA in Human Liver Cells. J. Biol. Chem..

[91] Demircan K., Bengtsson Y., Sun Q., Brange A., Vallon-Christersson J., Rijntjes E., Malmberg M., Saal L.H., Rydén L., Borg Å. (2021). Serum Selenium, Selenoprotein P and Glutathione Peroxidase 3 as Predictors of Mortality and Recurrence Following Breast Cancer Diagnosis: A Multicentre Cohort Study. Redox Biol..

[92] Mercer D.K. (2021). Selenium and Viral Infection: Are There Lessons for COVID-19. Br. J. Nutr..

[93] John R., Giudicessi B.A., Michael J., Pantalone D.W., Schneider K.L., Valentine S.E., Simoni J.M., Liu-Smith F., Pantalone D.W., Rood B.A. (2012). Iron Levels in Hepatocytes and Portal Tract Cells Predict Progression and Outcome of Patients with Advanced Chronic Hepatitis C. AIDS Behav..

[94] Milic S., Mikolasevic I., Orlic L., Devcic E., Starcevic-Cizmarevic N., Stimac D., Kapovic M., Ristic S. (2016). The Role of Iron and Iron Overload in Chronic Liver Disease. Med. Sci. Monit..

[95] Rostoker G., Vaziri N.D. (2017). Impact of Iatrogenic Iron Overload on the Course of Hepatitis C in the Dialysis Population: A Plea for Caution. Hemodial. Int..

[96] De Campos W.N., Massaro J.D., Cançado E.L.R., Wiezel C.E.V., Simões A.L., Teixeira A.C., De Souza F.F., Mendes-Junior C.T., Martinelli A.D.L.C., Donadi E.A. (2019). Comprehensive Analysis of HFE Gene in Hereditary Hemochromatosis and in Diseases Associated with Acquired Iron Overload. World J. Hepatol..

[97] Morris B.J., Willcox D.C., Donlon T.A., Willcox B.J. (2015). 2012 FOXO3: A major gene for human longevity-a mini-review. Gerontology.

[98] Chhabra R., Saha A., Chamani A., Schneider N., Nanjundan M., Shah R. (2020). Iron Pathways and Iron Chelation Approaches in Viral, Microbial, and Fungal Infections. Pharmaceuticals.

[99] Brem H., Stojadinovic O., Diegelmann R.F., Entero H., Lee B., Pastar I., Golinko M., Rosenberg H., Tomic-Canic M. (2007). Cholinergic Anti-Inflammatory Pathway Activity and High High Mobility Group Box-1 (HMGB1) Serum Levels in Patients with Rheumatoid Arthritis. Mol. Med..

[100] Wang Q., Liu Y., An D., Diao H., Xu W., He X., Sun R., Wei L., Li L. (2012). Regulation of Hepatitis C Virus Translation Initiation by Iron: Role of EIF3 and La Protein. Virus Res..

[101] Vela D. (2018). Low Hepcidin in Liver Fibrosis and Cirrhosis; A Tale of Progressive Disorder and a Case for a New Biochemical Marker. Mol. Med..

[102] Mansouri A., Gaou I., Fromenty B., Berson A., Letteron P., Degott C., Erlinger S., Pessayre D. (1997). “Premature MtDNA Deletions in Wilson’s Livers” Premature Oxidative Aging of Hepatic Mitochondrial DNA in Wilson’s Disease. Gastroenterology.

[103] Yu L., Liou I.W., Biggins S.W., Yeh M., Jalikis F., Chan L.N., Burkhead J. (2019). Copper Deficiency in Liver Diseases: A Case Series and Pathophysiological Considerations. Hepatol. Commun..

[104] Pauff S.M., Miller S.C. (2012). High Fructose Feeding Induces Copper Deficiency in SpragueDawley Rats: A Novel Mechanism for Obesity Related Fatty Liver. Bone.

[105] Jorquera F., Monte M.J., Guerra J., Sanchez-Campos S., Merayo J.A., Olcóz J.L., González-Gallego J., Marin J.J.G. (2005). Usefulness of Combined Measurement of Serum Bile Acids and Ferritin as Additional Prognostic Markers to Predict Failure to Reach Sustained Response to Antiviral Treatment in Chronic Hepatitis C. J. Gastroenterol. Hepatol..

[106] Escobedo-Monge M.F., Barrado E., Parodi-Román J., Escobedo-Monge M.A., Torres-Hinojal M.C., Marugán-Miguelsanz J.M. (2021). Copper and Copper/Zn Ratio in a Series of Children with Chronic Diseases: A Cross-Sectional Study. Nutrients.

[107] Sunada K., Minoshima M., Hashimoto K. (2012). Highly Efficient Antiviral and Antibacterial Activities of Solid-State Cuprous Compounds. J. Hazard. Mater..

[108] Deng S., Tjoa V., Fan H.M., Tan H.R., Sayle D.C., Olivo M., Mhaisalkar S., Wei J., Sow C.H. (2012). Reduced Graphene Oxide Conjugated Cu_2_O Nanowire Mesocrystals for High-Performance NO_2_ Gas Sensor. J. Am. Chem. Soc..

[109] Singh J., Srivastava M., Roychoudhury A., Lee D.W., Lee S.H., Malhotra B.D. (2013). Bienzyme-Functionalized Monodispersed Biocompatible Cuprous Oxide/Chitosan Nanocomposite Platform for Biomedical Application. J. Phys. Chem. B.

[110] Joshi S., Joshi M., Degani M.S. (2020). Tackling SARS-CoV-2: Proposed Targets and Repurposed Drugs. Future Med. Chem..

[111] Basu S. (2020). Non-Communicable Disease Management in Vulnerable Patients during COVID-19. Indian J. Med. Ethics.

[112] Guillin O.M., Vindry C., Ohlmann T., Chavatte L. (2019). Selenium, Selenoproteins and Viral Infection. Nutrients.

[113] Kieliszek M., Lipinski B. (2020). Selenium supplementation in the prevention of coronavirus infections (COVID-19). Med. Hypotheses.

[114] Martínez-González J., Varona S., Cañes L., Galán M., Briones A.M., Cachofeiro V., Rodríguez C. (2019). Emerging Roles of Lysyl Oxidases in the Cardiovascular System: New Concepts and Therapeutic Challenges. Biomolecules.

[115] Cherukuri S., Potla R., Sarkar J., Nurko S., Harris Z.L., Fox P.L. (2005). Unexpected Role of Ceruloplasmin in Intestinal Iron Absorption. Cell Metab..

[116] Cassat J.E., Skaar E.P. (2013). Iron in Infection and Immunity. Cell Host Microbe.

[117] Tarifeño-Saldivia E., Aguilar A., Contreras D., Mercado L., Morales-Lange B., Márquez K., Henríquez A., Riquelme-Vidal C., Boltana S. (2018). Iron Overload Is Associated with Oxidative Stress and Nutritional Immunity during Viral Infection in Fish. Front. Immunol..

[118] Bastin A., Shiri H., Zanganeh S., Fooladi S., Momeni Moghaddam M.A., Mehrabani M., Nematollahi M.H. (2021). Iron Chelator or Iron Supplement Consumption in COVID-19? The Role of Iron with Severity Infection. Biol. Trace Elem. Res..

[119] Woodby B., Arnold M.M., Valacchi G. (2021). SARS-CoV-2 Infection, COVID-19 Pathogenesis, and Exposure to Air Pollution: What Is the Connection?. Ann. N. Y. Acad. Sci..

[120] Shahid Z., Kalayanamitra R., McClafferty B., Kepko D., Ramgobin D., Patel R., Aggarwal C.S., Vunnam R., Sahu N., Bhatt D. (2020). COVID-19 and Older Adults: What We Know. J. Am. Geriatr. Soc..

[121] Choi S.H., Kim H.W., Kang J.M., Kim D.H., Cho E.Y. (2020). Epidemiology and Clinical Features of Coronavirus Disease 2019 in Children. Korean J. Pediatr..

[122] Kernan K.F., Carcillo J.A. (2017). Hyperferritinemia and Inflammation. Int. Immunol..

[123] Edeas M., Saleh J., Peyssonnaux C. (2020). Iron: Innocent Bystander or Vicious Culprit in COVID-19 Pathogenesis?. Int. J. Infect. Dis..

[124] Leermakers P.A., Remels A.H.V., Zonneveld M.I., Rouschop K.M.A., Schols A.M.W.J., Gosker H.R. (2020). Iron Deficiency-Induced Loss of Skeletal Muscle Mitochondrial Proteins and Respiratory Capacity; the Role of Mitophagy and Secretion of Mitochondria-Containing Vesicles. FASEB J..

[125] Ersöz A., Yılmaz T.E. (2021). The Association between Micronutrient and Hemogram Values and Prognostic Factors in COVID-19 Patients: A Single-Center Experience from Turkey. Int. J. Clin. Pract..

[126] Ganz T., Nemeth E. (2009). Iron Sequestration and Anemia of Inflammation. Semin. Hematol..

[127] Augustine L.F., Mullapudi V., Subramanian S., Kulkarni B. (2020). Infection-Iron Interaction during COVID-19 Pandemic: Time to Re-Design Iron Supplementation Programs. Med. Hypotheses.

[128] Habib H.M., Ibrahim S., Zaim A., Ibrahim W.H. (2020). The Role of Iron in the Pathogenesis of COVID-19 and Possible Treatment with Lactoferrin and Other Iron Chelators. Biomed. Pharmacother..

[129] Kell D.B., Heyden E.L., Pretorius E. (2020). The Biology of Lactoferrin, an Iron-Binding Protein That Can Help Defend Against Viruses and Bacteria. Front. Immunol..

[130] Tinkov A.A., Ajsuvakova O.P., Shehtman A.M., Boev V.M., Nikonorov A.A. (2012). Influence of Iron and Copper Consumption on Weight Gain and Oxidative Stress in Adipose Tissue of Wistar Rats. Interdiscip. Toxicol..

[131] Dubey P., Thakur V., Chattopadhyay M. (2020). Role of Minerals and Trace Elements in Diabetes and Insulin Resistance. Nutrients.

[132] Cruz K.J.C., De Oliveira A.R.S., Morais J.B.S., Severo J.S., Mendes P.M.V., Melo S.R.d., De Sousa G.S. (2018). Zinc and Insulin Resistance: Biochemical and Molecular Aspects. Biol. Trace Elem. Res..

[133] Tamaki M., Fujitani Y., Hara A., Uchida T., Tamura Y., Takeno K., Kawaguchi M., Watanabe T., Ogihara T., Fukunaka A. (2013). The Diabetes-Susceptible Gene SLC30A8/ZnT8 Regulates Hepatic Insulin Clearance. J. Clin. Investig..

[134] Ranasinghe P., Wathurapatha W.S., Galappatthy P., Katulanda P., Jayawardena R., Constantine G.R. (2018). Zinc Supplementation in Prediabetes: A Randomized Double-Blind Placebo-Controlled Clinical Trial. J. Diabetes.

[135] Sun Q., Van Dam R.M., Willett W.C., Hu F.B. (2009). Prospective Study of Zinc Intake and Risk of Type 2 Diabetes in Women. Diabetes Care.

[136] Schomburg L. (2021). Selenium Deficiency Due to Diet, Pregnancy, Severe Illness, or COVID-19—A Preventable Trigger for Autoimmune Disease. Int. J. Mol. Sci..

[137] Roden M., Shulman G.I. (2019). The Integrative Biology of Type 2 Diabetes. Nature.

[138] Steinbrenner H., Duntas L.H., Rayman M.P. (2022). The Role of Selenium in Type-2 Diabetes Mellitus and Its Metabolic Comorbidities. Redox Biol..

[139] Kim J., Chung H.S., Choi M.K., Roh Y.K., Yoo H.J., Park J.H., Kim D.S., Yu J.M., Moon S. (2019). Association between Serum Selenium Level and the Presence of Diabetes Mellitus: A Meta-Analysis of Observational Studies. Diabetes Metab. J..

[140] Stranges S., Galletti F., Farinaro E., D’Elia L., Russo O., Iacone R., Capasso C., Carginale V., De Luca V., Della Valle E. (2011). Associations of Selenium Status with Cardiometabolic Risk Factors: An 8-Year Follow-up Analysis of the Olivetti Heart Study. Atherosclerosis.

[141] Alghobashy A.A., Alkholy U.M., Talat M.A., Abdalmonem N., Zaki A., Ahmed I.A., Mohamed R.H. (2018). Trace Elements and Oxidative Stress in Children with Type 1 Diabetes Mellitus. Diabetes Metab. Syndr. Obes. Targets Ther..

[142] Vinceti M., Filippini T., Wise L.A., Rothman K.J. (2021). A Systematic Review and Dose-Response Meta-Analysis of Exposure to Environmental Selenium and the Risk of Type 2 Diabetes in Nonexperimental Studies. Environ. Res..

[143] Liao X.L., Wang Z.H., Liang X.N., Liang J., Wei X.B., Wang S.H., Guo W.X. (2020). The Association of Circulating Selenium Concentrations with Diabetes Mellitus. Diabetes Metab. Syndr. Obes. Targets Ther..

[144] Cheng Z., Li Y., Young J.L., Cheng N., Yang C., Papandonatos G.D., Kelsey K.T., Wise J.P., Shi K., Zheng T. (2022). Long-Term Association of Serum Selenium Levels and the Diabetes Risk: Findings from a Case-Control Study Nested in the Prospective Jinchang Cohort. Sci. Total Environ..

[145] Panel E., Nda A. (2015). Scientific Opinion on Dietary Reference Values for Magnesium. EFSA J..

[146] Lobionda S., Sittipo P., Kwon H.Y., Lee Y.K. (2019). The Role of Gut Microbiota in Intestinal Inflammation with Respect to Diet and Extrinsic Stressors. Microorganisms.

[147] Gill J.M.R. (2012). Type 2 Diabetes. Nurs. Made Incred. Easy.

[148] Fang X., Han H., Li M., Liang C., Fan Z., Aaseth J., He J., Montgomery S., Cao Y. (2016). Dose-Response Relationship between Dietary Magnesium Intake and Risk of Type 2 Diabetes Mellitus: A Systematic Review and Meta-Regression Analysis of Prospective Cohort Studies. Nutrients.

[149] Kostov K. (2019). Effects of Magnesium Deficiency on Mechanisms of Insulin Resistance in Type 2 Diabetes: Focusing on the Processes of Insulin Secretion and Signaling. Int. J. Mol. Sci..

[150] Apell H.J., Hitzler T., Schreiber G. (2017). Modulation of the Na,K-ATPase by Magnesium Ions. Biochemistry.

[151] Zhao B., Zeng L., Zhao J., Wu Q., Dong Y., Zou F., Gan L., Wei Y., Zhang W. (2020). Association of Magnesium Intake with Type 2 Diabetes and Total Stroke: An Updated Systematic Review and Meta-Analysis. BMJ Open.

[152] Jayawardena R., Ranasinghe P., Galappatthy P., Malkanthi R.L., Constantine G.R., Katulanda P. (2012). Effects of Zinc Supplementation on Diabetes Mellitus: A Systematic Review and Meta-Analysis. Diabetol. Metab. Syndr..

[153] ClinicalTrials.gov. https://clinicaltrials.gov/ct2/show/NCT05320510?term=selenium&cond=diabetes+type+2&draw=2&rank=1.

[154] Saeed H., Haj S., Qasim B. (2019). Estimation of Magnesium Level in Type 2 Diabetes Mellitus and Its Correlation with HbA1c Level. Endocrinol. Diabetes Metab..

[155] ClinicalTrials.gov. https://clinicaltrials.gov/ct2/show/NCT03002545?term=magnesium&cond=type+2+diabetes&draw=2&rank=3.

[156] ClinicalTrials.gov. https://clinicaltrials.gov/ct2/show/NCT05033054?term=magnesium&cond=type+2+diabetes&draw=2&rank=6.

[157] ClinicalTrials.gov. https://clinicaltrials.gov/ct2/results?cond=COVID-19&term=selenium&cntry=&state=&city=&dist=.

[158] ClinicalTrials.gov. https://clinicaltrials.gov/ct2/show/NCT04877509?term=selenium&cond=COVID-19&draw=2&rank=2.

[159] ClinicalTrials.gov. https://clinicaltrials.gov/ct2/show/NCT04941703?term=magnesium&cond=covid19&draw=2&rank=1.

[160] ClinicalTrials.gov. https://clinicaltrials.gov/ct2/show/NCT04716985?term=magnesium&cond=covid19&draw=2&rank=3.

[161] ClinicalTrials.gov. https://clinicaltrials.gov/ct2/show/NCT04641195?term=zinc&cond=COVID-19&draw=2&rank=1.

[162] ClinicalTrials.gov. https://clinicaltrials.gov/ct2/show/NCT04370782?term=zinc&cond=COVID-19&draw=2&rank=2.

[163] ClinicalTrials.gov. https://clinicaltrials.gov/ct2/show/NCT04558424?term=zinc&cond=COVID-19&draw=2&rank=3.

[164] ClinicalTrials.gov. https://clinicaltrials.gov/ct2/show/NCT04542993?term=zinc&cond=COVID-19&draw=2&rank=6.

[165] ClinicalTrials.gov. https://clinicaltrials.gov/ct2/show/NCT04072822?term=zinc&cond=hepatitis&draw=2&rank=1.

[166] ClinicalTrials.gov. https://clinicaltrials.gov/ct2/show/NCT01809132?term=zinc&cond=hepatitis&draw=2&rank=2.

[167] ClinicalTrials.gov. https://clinicaltrials.gov/ct2/show/NCT01355107?term=selenium&cond=hepatitis&draw=2&rank=1.

[168] ClinicalTrials.gov. https://clinicaltrials.gov/ct2/show/NCT03349008?term=magnesium&cond=hepatitis&draw=2&rank=1.

[169] ClinicalTrials.gov. https://clinicaltrials.gov/ct2/show/NCT03166280?term=NCT03166280&draw=2&rank=1.

[170] ClinicalTrials.gov. https://clinicaltrials.gov/ct2/show/NCT02744560?term=iron&cond=hepatitis&draw=2&rank=4.

